# Using the LACE index to predict hospital readmissions in congestive heart failure patients

**DOI:** 10.1186/1471-2261-14-97

**Published:** 2014-08-07

**Authors:** Hao Wang, Richard D Robinson, Carlos Johnson, Nestor R Zenarosa, Rani D Jayswal, Joshua Keithley, Kathleen A Delaney

**Affiliations:** 1 Department of Emergency Medicine, Integrative Emergency Service, John Peter Smith Health Network, 1500 S. Main St, 76104 Fort Worth, TX, USA

## Abstract

**Background:**

The LACE index has been used to predict the risk of unplanned readmission within 30 days after hospital discharge in both medical and surgical patients. The aim of this study is to validate the accuracy of using the LACE index in CHF patients.

**Methods:**

This was a retrospective study. The LACE index score was calculated on each patient who was admitted to hospital due to an acute CHF exacerbation. Operational and clinical variables were collected from patients including basic clinical characteristics, length of hospitalization, comorbidities, number of previous ED visits in the past 6 months before the index admission, and the number of post discharge ED revisits at 30, 60, and 90 days. All variables were analyzed by multivariate logistic regression to determine the association between clinical variables and the hospital unplanned readmissions. C-statistic was used to discriminate those patients with high risk of readmissions.

**Results:**

Of the 253 patients included in the study, 24.50% (62/253) experienced unplanned readmission to hospital within 30 days after discharge. The LACE index was slightly higher in patients readmitted versus patients not readmitted (12.17 ± 2.22 versus 11.80 ± 1.92, *p* = 0.199). Adjusted odds ratios based on logistic regression of all clinical variables showed only the number of previous ED visits (OR 1.79, 95% CI 1.30-2.47, p < 0.001), history of myocardial infarction (OR 2.51, 95% CI 1.02-6.21, *p* = 0.045), and history of peripheral vascular disease (OR 10.75, 95% CI 1.52-75.73, *p* = 0.017) increased the risk of unplanned readmission within 30 days of hospital discharge. However, patients with high LACE scores (≥10) had a significantly higher rate of ED revisits (15.04% vs 0%) within 30 days from the index discharge than those with low LACE scores (*p* = 0.030).

**Conclusion:**

The LACE index may not accurately predict unplanned readmissions within 30 days from hospital discharge in CHF patients. The LACE high risk index may have utility as a screening tool to predict high risk ED revisits after hospital discharge.

## Background

Readmission is considered a second admission to hospital within a short period of time (usually within 30 days) from hospital discharge and has been considered both costly and an indication of poor health care quality delivery [1,2]. A recent study reported 30 days hospital readmission of approximately 20% among all Medicare patients with an estimated cost of 17.4 billion US dollars [3]. In order to properly use and reduce the significant healthcare cost, a number of interventions designed to prevent and reduce hospital readmissions were reported by different studies. However, the success of these studies was largely dependent on proper identification of patients at high risk for readmission [4-6]. Several scoring systems have been reported to predict unplanned readmissions and the LACE index is one of the most commonly used systems in the US [1,7-9].

The LACE index has been used to predict the risk of unplanned readmission or death within 30 days after hospital discharge in both medical and surgical patients. It includes the length of hospitalization stay (“L”), acuity of the admission (“A”), comorbidities of patients (“C”), and emergency department use of patients (“E”). Studies of the LACE index showed that higher LACE scores predicted higher patient readmissions (see detail in Table 1). In general, the LACE index score of greater than ten is considered patients at high risk for unplanned hospital readmissions [8]. However, the results of the LACE index in predicting readmissions were controversial in different studies, especially lack of validation if applied to a particular patient population [10,11].

**Table 1:** Detail of LACE score salculation

**LACE score calculation table**		
**L-Score**	**Length of stay (days)**	**Score**
	1	1
	2	2
	3	3
	4-6	4
	7-13	**5**
	14 or more	**7**
**A-Score**	**Acuity of Admission**	Score
	Yes	3
	No	0
**C-Score**	**Comorbidities**	Score
If the total score is between 0 and 3, C-score equals to the total score, if the total score is 4 or higher, C-score is 5	Previous myocardial infarction	+1
	Cerebrovascular disease	+1
	Peripheral vascular disease	+1
	Diabetes without complications	+1
	Congestive heart failure	+2
	Diabetes with end organ damage	+2
	Chronic pulmonary disease	+2
	Mild liver or renal disease	+2
	Any tumor (including lymphoma or leukemia)	+2
	Dementia	+3
	Connective tissue disease	+3
	AIDS	+4
	Moderate or severe liver or renal disease	+4
	Metastatic solid tumor	+6
	TOTAL	
**E-score**	**Emergency department visits**	Score
	How many times has the patient visited an emergency department in the six months prior to admission (not including the emergency department visit immediately preceding the current admission)?	Score equal to the same number of ED visits if the number of ED visits less than 4. If the number of ED visits more than 4 (including 4), E-score is 4.
Total LACE score = L-score + A-score + C-score + E-score 0–4 Low, 5–9 Moderate, > 9 High Risk		

Modified and Adapted from *qio.ipro.org/wp-content/uploads/2013/01/****LACE*** *_* ***tool*** *NEW.doc.*

Congestive heart failure (CHF) is one of the most common and severe diseases seen in the Emergency Department (ED) and is associated with very high hospital admissions and readmissions [12,13]. Previous studies in CHF showed readmissions might be associated with length of hospitalization, medical comorbidities, frequent ED revisits, low literacy skills, and severity of illness [14-16]. In order to reduce high readmissions, it is suggested that high risk CHF patients need to be identified earlier and be linked to some intervention programs upon discharge [13,17].

Therefore, the aim of this observational study was to externally validate the accuracy of using the LACE index to predict 30 days readmissions in CHF patients. Our secondary goal was to determine the potential independent risk factors that are predictive of readmission if the LACE index was determined unreliable.

## Methods

Study Design and Population: This was a retrospective study of adult patients who presented to an urban publicly funded hospital ED with CHF exacerbations between June 2012 and June 2013. The study population included patients greater than 18 years of age. All patients were admitted to hospital from the ED due to CHF exacerbation. Patients receiving a discharge diagnosis of CHF based on criteria from the 9^th^ edition of international classification of disease (ICD-9) code were included. The diagnosis of CHF was confirmed by cardiology consult notes, ICD-9 code discharge diagnosis, or recent echocardiography report. Recent echocardiography was defined as a study performed at either the index admission or within 6 months. For patients admitted more than once during the study period, only the initial admission was included. Study excluded patients who died in hospital or whose primary diagnosis was not CHF exacerbation. Study also excluded patients that were not admitted directly from ED due to an extreme small sample size (only 5 patients) likely causing significant bias. Hospital readmission was defined as all cause patient readmission from the ED for the second time within 30 days of the index admission. It is assumed that patient readmissions had not been arranged or planned if they presented directly to the ED prior to hospital readmission. This study was approved by the local John Peter Smith Health Network Institutional Review Board.

Clinical variables and data collection: Patient demographic data and their medical history records were reviewed using the electronic health record. Variables including age, gender, ethnicity, the number of previous ED visit within 6 months, the length of hospitalization stay (LOS), and the Charlson comorbidity index (CCI) were collected. The LACE index was then calculated as reported in previous studies [14-16]. Thirty days readmissions, mortality, and post discharge ED visits were also obtained from the electronic health record.

Statistics: Univariate comparisons between patients that were readmitted to hospital within 30 days versus those not readmitted were analyzed. The student *t* test was used for continuous data and the χ^2^ test was used for categorical data. To exclude potential confounding factors, all variables were then analyzed using multivariate logistic regression. C-statistic (area under the receiver operating characteristics) with 95% confidence intervals (CI) was used to assess the discriminatory power of the model and compare goodness of fit for logistic regression. A value of 0.5 indicates the model is no better than chance for making a prediction. A value of 0.7 or higher is considered statistically significant to identify a difference between groups. All other tests were two-sided with *p* value less than 0.05 considered statistically significant. Stata 12.0 statistical software (Stata Corp, College Station, TX, USA) was used for statistical data analysis.

## Results

### The LACE index may not accurately predict unplanned readmissions

Between June 2012 and June 2013, a total of 343 patients were admitted to hospital with the primary admission diagnoses of CHF exacerbations. Among all these 343 patients, 5 patients were directly admitted from outside ED and the other 85 patients were admitted to hospital multiple times with CHF exacerbations. Therefore, a total of 253 patients were included in this study. The rate of unplanned readmission to hospital within 30 days of index discharge for this group was 24.50% (62/253). The average Charlson comorbidity index (CCI) score was 4.56 ± 0.98 and average LACE index score was 11.89 ± 2.00. Considering a higher LACE index (LACE ≥10) indicates higher unplanned readmissions, patients were divided into two groups based on their LACE index scores. There were no statistical significant difference on patient basic characteristics between these two groups including age, gender, and ethnicity (p > 0.05). However, our results also show no significant difference in readmissions between these two groups as well (High LACE group: 24.34% versus Low LACE group: 25.93%, p = 0.856). This indicates that a value of 10 may not accurately differentiate the low limit index score in terms of predicting unplanned readmissions in our CHF patients. In order to determine whether the LACE index itself is a reliable clinical decision tool and what is an accurate low limit index score above which the predictability of readmissions among high risk patients is improved, patients were stratified into two groups based on whether or not they experienced unplanned readmissions within 30 days of index discharge. The results of our study show the LACE index is slightly higher in patients that experienced readmissions versus those that did not (12.17 ± 2.22 versus 11.80 ± 1.92, p = 0.199).The LACE index in both groups was higher than 10 and no statistically significant difference was reached (Table 2). In addition, the C-statistic of logistic regression for the LACE index was 0.5610 (95% CI: 0.4771-0.6447) which was again not discriminative for predicting readmissions in the study population regardless whether or not patients achieved a high risk LACE index. Therefore, our results suggest that the LACE index may not accurately predict for unplanned readmissions in CHF patients.

**Table 2:** The basic characteristics of hospitalized CHF patients in groups of whether experienced readmissions

	**Readmission n = 62**	**No readmission n = 191**	**p**
LACE index ± SD	12.17 ± 2.22	11.80 ± 1.92	0.199
Age, mean year ± SD	57.67 ± 11.68	56.17 ± 11.43	0.375
Age group, %			
<65 years	75.81	79.58	0.529
≥65 years	24.19	20.42	
Male Gender, %	62.90	61.78	0.874
Ethnicity and race, %			
White	22.58	29.32	0.510
Africa American	59.68	49.74	
Hispanic	16.13	17.28	
Others	1.61	3.66	
Length of index hospitalization stay, day ± SD	5.33 ± 3.32	5.90 ± 3.88	0.307
Number of ED visits 6 months before the index admission ± SD	0.80 ± 1.29	0.34 ± 0.73	<0.001
Charlson comorbidity index ± SD	4.62 ± 0.87	4.53 ± 1.01	0.532
Mode of arrival by Ambulance	35.48% (22/62)	38.30% (72/188)	0.692
No primary care physician/unknown	51.61% (32/62)	47.64% (91/191)	0.587
Psychiatric problems	17.74% (11/62)	22.51% (43/191)	0.426
Insurance type			
Charity program	27.42% (17/62)	19.90% (38/191)	0.611
Commercial insurance	0	1.57% (3/191)	
Medicaid	17.74% (11/62)	24.61% (47/191)	
Medicare	27.42% (17/62)	23.56% (45/191)	
Self pay	20.97% (13/62)	23.04% (44/191)	
Unknown	6.45% (4/62)	7.33% (14/191)	

Frequent ED visits before the index admission and history of myocardial infarction or peripheral vascular disease might increase the risk of readmission within 30 days from index discharge in CHF patients.

All clinical variables were analyzed to determine potential independent risk factors that could predict readmission more accurately than the LACE index. In order to avoid confounding factors, a multivariate logistic regression model was used for variable analysis. Our results showed only three clinical variables demonstrate significant differences between patients readmitted within 30 days of discharge versus those not readmitted. These independent risk factors included (1) the number of ED visits in the past 6 months before the index admission (adjusted OR = 1.79), (2) history of myocardial infarction (MI, adjusted OR = 2.51), and (3) history of peripheral vascular diseases (PVD, adjusted OR = 10.75). Table 3 shows the adjusted odds ratios of clinical variables between the readmission versus non-readmission groups. Our findings suggest frequent prior ED visits and history of MI or PVD might increase the risk of readmissions within 30 days from hospital discharge in CHF patients.

**Table 3:** Adjusted odds ratios of clinical variables by multivariate logistic regression between patients readmitted within 30 days from discharge and those without

	**OR**	**95% CI**	**p-value**
Age	1.01	0.98-1.04	0.245
Gender (Male)	1.25	0.64-2.44	0.508
Length of index hospitalization stay	0.92	0.83-1.02	0.141
LACE ≥10	0.45	0.09-2.07	0.309
Charlson comorbidity index	1.21	0.69-2.13	0.498
Number of ED visits 6 months before the index admission	1.79	1.30-2.47	<0.001
History of myocardial infarction	2.51	1.02-6.21	0.045
History of peripheral vascular disease	10.75	1.52-75.73	0.017

### LACE index can predict ED revisits more accurately than it predicts unplanned readmissions

**Figure 1:**
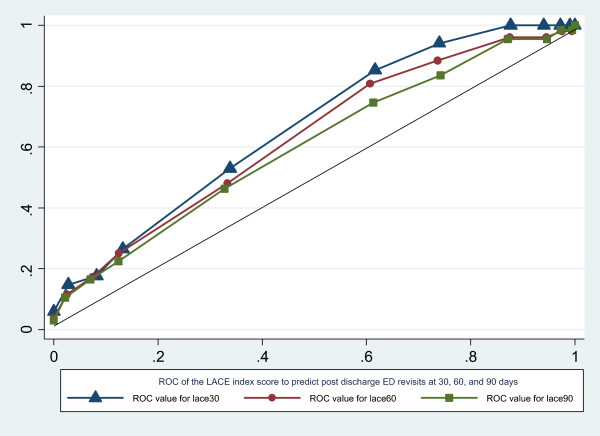
**Showed ROC of the LACE index score to predict post discharge ED revisits at 30, 60, and 90 days.** The C-statistic of the logistic regression for the LACE index to predict post discharge 30 days ED revisits is 0.6637 (95% CI: 0.5750-0.7523), 60 days post discharge ED revisits is 0.6307 (95% CI: 0.5485-0.7128), and that of the post discharge 90 days ED revisits is 0.6029 (95% CI: 0.5248-0.6809) which was more accurate than predicting hospital readmissions at the same time period in the study population though still not overwhelming (C-statistic <0.7).

Patients were followed up after 30, 60, and 90 days from the index discharge. Unplanned readmissions, deaths, and post discharge ED revisits were reviewed. We analyzed the role of the LACE index in the prediction of both unplanned hospital readmissions and post discharge ED revisits. Our results showed high risk patients (LACE ≥10) had slightly lower unplanned readmissions within 30 days from index discharge, however no significant statistical difference was reached between groups. Only one patient died within 30 days of hospital discharge and this patient fell into the high risk LACE index group. In addition, when extending unplanned readmissions analysis to 60 and 90 days, the LACE index again demonstrated poor prediction of readmissions. When ED revisits after the index hospital discharge were evaluated, our results show that patients with a high LACE score (≥10) had a higher rate of ED revisits (15.04%) within 30 days from index discharge, whereas, no patient with a low LACE score visited the ED within 30 days. The results were also consistent when extended up to 90 days from index discharge (Table 4). Using the LACE index to predict likelihood of ED revisits at 30, 60, and 90 days post discharge the odds ratios were 1.42, 1.29, and 1.23 separately (p < 0.01). The C-statistics associated with the LACE index were 0.66, 0.63, and 0.60 respectively (Figure 1). Taken together, our study shows the LACE index can predict ED revisits more accurately than unplanned readmissions after the index discharge.

**Table 4:** The role of high risk LACE index on predicting unplanned readmissions and Emergency revisits after index discharge

	**High risk for readmission N = 226**	**Low risk for readmission N = 27**	**OR (95% CI)**	**P value**
Within 30 days after index discharge				
Hospital unplanned readmission, %	24.34	25.93	0.91 (0.36-2.28)	0.856
ED revisits, %	15.04	0	N/A	0.030
Mortality, %	0.44	0	N/A	0.729
Within 60 days after index discharge				
Hospital unplanned readmission, %	30.97	40.74	0.65 (0.28-1.47)	0.304
ED revisits, %	22.12	7.42	3.55 (0.81-15.50)	0.074
Mortality, %	0.44	0	N/A	0.729
Within 90 days after index discharge				
Hospital unplanned readmission, %	41.05	48.15	0.75 (0.33-1.67)	0.486
ED revisits, %	28.32	11.11	3.16 (0.91-10.86)	0.055
Mortality, %	0.44	0	N/A	0.726

## Discussion

Unplanned hospital readmissions are considered a marker of the quality of hospital care based on the underlying reason that return visits were related to premature discharge and inadequate treatment [18,19]. This occurs commonly in the CHF population [20]. The LACE index is an easy to use scoring tool that was reported by several studies to be accurate for predicting the risk of early death or unplanned readmission after discharge from hospital [17,21,22]. However, other studies showed poor performance of the LACE index in different patient populations [16,23,24]. Our results also show that use of the LACE index to identify high risk CHF patients for readmissions is not reliable.

Our study population had a high Charlson comorbidity index score (mean CCI = 4.56) resulting in a higher average LACE score (mean LACE score = 11.89). However, the population examined whereby the LACE index was initially derived had a mean CCI score of 0.5 and a mean LACE score of 6.0 which was much lower than our study population [14]. This may, in part, explain the inconsistency of using the LACE index to predict readmissions in our study. In addition, our study population had relatively high psychosocial risks including transportation challenges, active psychiatric problems, lacking primary care physicians, less commercial insurance coverage, or no insurance which could lead to frequent ED visits. This phenomenon not only occurred in patients with frequent readmissions, it also occurred commonly in patients with no readmissions showing the high consistency in this study population (Table 2). Similar results from other studies showed the high occurrence of medication noncompliance, a lack of primary care follow up, and/or a lack of family support placed patients into a high risk group more likely to develop chronic disease exacerbations resulting in more frequent hospital readmissions [25-27]. Taken together, it is noted that due to significant differences in study populations, the LACE index may not reliably predict hospital readmissions accurately.

An independent risk factor that demonstrated reliable predictability of readmissions in our study was number of ED visits prior to the index admission. This suggests that frequent ED visits may result in a higher frequency of hospital readmissions. This could be related to a higher burden of chronic illness among these patients. Patients having chronic medical conditions may experience a higher frequency of ED visits, higher rates of hospital admissions, and more lengthy hospital stays [27,28]. It could also be attributed to the extreme age reported in some studies [28,29]. Our study results showed length of hospital stay, age, and CCI score had no significant difference regardless as to whether patients were readmitted or not. Further review of chronic illnesses in the study population showed that patients with a history of MI or PVD tended to have more hospital readmissions. Similar results are found within Medicare data reporting comorbidities including history of diabetes, MI, PVD, and stroke could predict a higher risk of readmissions in CHF patients [30]. Previous studies found MI and CHF were the two most common diseases associated with high admissions and readmissions [31]. The coexistence of these two chronic conditions is high [32]. Our study confirmed the hypothesis and showed that CHF patients with history of MI experienced higher readmissions but did not discriminate as to whether or not MI was a consequence of CHF.

To the best of our knowledge, there is no previously published study that investigates the link between the LACE index and post index discharge ED revisits. Our study found that the LACE index can predict post discharge ED revisits more accurately than it predicts hospital readmissions though this predictability is not overwhelming (c < 0.7). A high LACE index predicts a high risk of post discharge ED visits when patients are followed up to 90 days post index discharge. Additionally patients with a low LACE index experienced no ED revisits within 30 days post index discharge. Our study also showed CHF patients with history of prior frequent ED visits are highly likely to have the same pattern of frequent ED revisits during the post index discharge period. This could be due, at least partially, to the high psychosocial risks and associated comorbidities in our study population. These patients tend to use the ED as their primary resource for health care. In addition, given the fact of other comorbidities affecting hospital readmissions indicates its complexity and multifactory. Since the LACE index is a user friendly tool, it is practical to screen CHF patients before hospital discharge to identify relatively high risk patients. With limited health care resources at their disposable, these CHF patients with a high risk LACE index can be prioritized to services linking them to community intervention programs. Several CHF intervention programs are now in place in our community providing an alternate resource for these patients and curbing ED overuse for health maintenance needs among them.

### Limitation

This is a retrospective study from a single urban tertiary care publicly funded hospital with a special patient population. Retrospective methodology limits its applicability in a way that includes bias regarding the accuracy of information, potential selection bias due to one institutional database, incomplete follow up data, and incomplete inter-subject data for analysis. In addition, as this study examines data associated only with patients admitted from the ED and excludes patients admitted from other primary care services this might also lead to patient selection bias. Second, all readmissions were reviewed and collected from hospital electronic health records. Patients that may have been admitted to other hospitals were not included in this study. This may contribute to incomplete follow up data. We did monitor other measures during the patient post-discharge period, such as follow up visits with primary care physicians and ED revisits. With many high risk psychosocial patients in our study population a certain number of them were lost to follow up which may also lead to incomplete and/or inaccurate analysis of data. A multicenter prospective study is required to more accurately identify CHF patients at high risk of hospital readmission and ED revisits and to further validate the results of this study.

## Conclusion

In conclusion, our study shows application of the LACE index to identify CHF patients at high risk for unplanned readmissions within 30 days of discharge may not be reliable. Better predictors of high risk readmissions among CHF patients may be 1) number of previous ED visits, 2) history of MI, 3) and/or history of PVD. A LACE high risk index may have the best application in predicting the likelihood of ED revisits in the post index discharge period and therefore be of particular interest in linking these patients to alternate community healthcare services thereby curbing ED overuse among this population.

## Competing interests

The authors declare no competing interests.

## Authors' contributions

HW conceived of the study, participated in the study design, data collection, statistical analysis, data interpretation, and drafted the manuscript. RDR participated in the study design, data interpretation, coordination, and helped to draft the manuscript. CJ participated data collection and interpretation. NRZ participated in the design of the study, coordination, and helped to draft the manuscript. RDJ participated in the data collection and performed the statistical analysis. JK participated in the data collection and helped to draft the manuscript. KAD participated in the study design and helped to draft the manuscript. All authors read and approved the final manuscript.

## Pre-publication history

The pre-publication history for this paper can be accessed here:

http://www.biomedcentral.com/1471-2261/14/97/prepub
